# Identification and genetic diversity analysis of broomrape in Xinjiang, China

**DOI:** 10.1007/s11033-023-09203-9

**Published:** 2024-02-23

**Authors:** Xuekun Zhang, Juan Du, Panpan Wang, Peng Wang, Zheng Liu, Zhaoqun Yao, Sifeng Zhao, Hui Xi

**Affiliations:** 1 College of Agriculture/Key Laboratory of Oasis Agricultural Pest Management and Plant Protection Resources Utilization, Xinjiang Uygur Autonomous Region, Shihezi University, Shihezi, 832003 Xinjiang China; 2 Xinjiang Academy of Agricultural Reclamation Sciences, Shihezi, 832000 Xinjiang China

**Keywords:** Broomrape, Phylogenetic analysis, Genetic diversity, ISSR

## Abstract

**Background:**

As a holoparasitic weed, broomrape has seriously threatened the production of economically important crops, such as melon, watermelon, processed tomato, and sunflower, in Xinjiang in recent years. However, the distribution and genetic diversity of broomrape populations in Xinjiang are not clear at present, which hinders their prevention and control. The purpose of this study was to identify the main species and the genetic differentiation structure of the broomrape population in Xinjiang.

**Methods and results:**

In the present study, 93 samples from different geographic regions of Xinjiang were collected to identify the species based on ITS and plastid *rps*2 regions, and the samples were also used to analyze the genetic diversity based on ISSR markers. The results showed that broomrape is not monophyletic in Xinjiang and consists of two major clades (*Orobanche* cf. *aegyptiaca* and *O. cernua*) and three subclades (*O.* cf. *aegyptiaca* var. *tch*, *O.* cf. *aegyptiaca* var. *klz*, and *O. cernua.*var. *alt*) based on phylogenetic analysis. Furthermore, the results of the genetic diversity analysis indicated that the average polymorphic information content and marker index were high values of 0.58 and 7.38, respectively, showing the efficiency of the ISSR markers in detecting polymorphism among the broomrape population studied. Additionally, the 11 selected primers produced 154 repeatable polymorphic bands, of which 150 were polymorphic. The genetic diversity of the samples was 37.19% within populations and 62.81% among the populations, indicating that the main genetic differentiation occurred among the populations. There was less gene exchange between populations, with a gene flow index (Nm) of 0.2961 (< 1). The UPGMA dendrogram indicated that most populations with similar geographical conditions and hosts were clustered first, and then all samples were separated into two major groups and seven subclusters.

**Conclusion:**

The broomrapes are mainly *O*. cf. *aegyptiaca* and *O. cernua* in Xinjiang, which were separated into two major groups and seven subclusters based on ISSR markers. Our results provide a theoretical basis for breeding broomrape-resistant varieties.

**Supplementary Information:**

The online version contains supplementary material available at 10.1007/s11033-023-09203-9.

## Introduction

Parasitic weeds pose a serious threat to agriculture, causing devastating damage to agricultural crops throughout the world. The Orobanchaceae family, known as broomrape, is a holoparasitic plant with no chlorophyll, and nutrients, minerals, and water are obtained from the host by haustoria [1]. Broomrape can severely retard the growth and productivity of its host plants and cause crop losses that reach billions of dollars annually [2, 3]. At present, some methods have been applied in the field to control broomrape, such as cultural practices, suicidal germination, activation of systemic acquired resistance, biocontrol, and herbicides [4, 5]. However, the reproductive capacity of broomrape is very strong; each plant can produce 100 thousand seeds [4], of which *O. aegyptiaca* has a stronger reproductive capacity, producing 500 thousand to 3 million seeds per plant and possibly remaining dormant for more than 15 years [6, 7], which makes broomrape control extremely difficult.

The most efficient and economical method for controlling broomrape resistance is crop breeding [8, 9]. At present, no broad-spectrum resistant varieties against broomrape have been found, and existing resistant hosts mainly show resistance to specific races; for example, the sunflower P-96 shows dominant resistance to broomrape race E and recessive resistance to the very new race F [10], and the sunflower *HaOr7* mainly controls the Spanish broomrape race F populations [11]. It is important to clarify the species and genetic diversity of broomrape populations to cultivate varieties that are resistant to broomrape. However, the species and population diversity of broomrape in Xinjiang are currently unclear. It has been reported that Orobanchaceae has 19 species in Xinjiang, of which 15 species are *O.* L [12]. Furthermore, Orobanchaceae is a morphologically diverse family with a wide range of hosts [1, 4], and many morphological characteristics useful for species identification have been lost with the evolution of the species [13, 14].

The study of natural broomrape populations can help us to understand gene pool dynamics, population size structure, geographical distribution, environmental adaptation, and origin. Morphological characteristics are limited to application diversity studies because of their variation with environmental changes, estimation errors, and reduced number of characteristic features available. Previously, several approaches have been used to identify the species and populations. Among these markers, plastid sequences, internal transcribed spacer (ITS) sequences of nuclear ribosomal DNA, RAPD, and ISSR markers have been used to identify species and analyze genetic diversity [15, 16]. Clarifying their species and distribution is conducive to cultivating varieties resistant to broomrape.

To clarify the species and genetic diversity of broomrape in Xinjiang, 93 samples from different geographic regions of Xinjiang were collected to identify the species based on nuclear ITS and plastid *rps*2 regions. The genetic diversity was analyzed based on inter-simple sequence repeat (ISSR) markers. Our results provide a theoretical basis for the prevention, control, and breeding of broomrape-resistant varieties.

## Materials and methods

### Samples collected

The plants and seeds of 93 broomrape plants were collected from different regions of Xinjiang during the crop growth stage (Table 1). The hosts mainly includes processed tomato, melon, seed watermelon and sunflower, stored in a refrigerator at - 70 °C before use.

**Table 1:** List of broomrape plants selected for this study

Population	Location	Sample number	Host	longitude and latitude of sample center location
Second division	Regiment 21	P48, P73, P74, P75, P76	Processing tomato	E86°18′58.03″, N42°9′30.57″
	Regiment 22	P52, P65, P79, P80, P81, P82	Processing tomato	E86°32′45.42″, N42°10′41.53″
	Regiment 25	P49, P63, P64, P66, P69, P70, P83, P84, P85, P86, P87	Processing tomato	E86°39′38.16″, N41°59′49.12″
	Regiment 27	P47, P56, P77, P78	Processing tomato	E86°33′46.06″, N42°0′25.45″
Kashi city	Ba Chu county	P50	Processing tomato	E78°34′51.85″, N39°48′5.10″
	Ba Chu county	P51	Pumpkin	E78°34′51.72″, N39°48′4.211″
	Ba Chu county	P55	Cowpea	E78°34′48.10″, N39°48′3.61″
	Ba Chu county	P61	Tomato	E78°34′43.19″, N39°47′59.42″
	Ba Chu county	P67	Chilli	E78°34′47.84″, N39°47′59.82″
	Jia Shi county	P57, P59, P72	Melon	E76°44′58.17″, N39°30′53.44″
	Shu Fu county	P58, P62	Melon	E75°52′8.91″, N39°22′11.53″
Fourth division	62 Regiment	P3, P8, P9, P10	Processing tomato	E80°28′3.74″, N44°9′58.41″
	62 Regiment	P18, P19, P21, P24	Seed watermelon	E80°28′2.76″, N44°9′57.79″
Seventh division	Kui Tun city	P28, P31, P32, P33, P34	Processing tomato	E84°57′43.47″, N44°25′52.33″
Eighth division	145 Regiment	P1	Sunflower	E86°0′36.39″, N44°20′24.75″
	145 Regiment	P2	Processing tomato	E86°0′36.86″, N44°20′39.56″
	Xinjiang Academy of Agricultural and Reclamation Science	P4, P5	Sunflower	E86°0′22.42″, N44°18′42.14″
	88 Regiment	P20	Oil sunflower	E81°0′7.39″, N44°58′20.93″
	Tuan Jie farm	P16	Processing tomato	E83°28′47.79″, N46°31′30.71″
Ninth division	163 Regiment	P6、P7、P23	Seed watermelon	E82°54′3.02″, N46°44′23.13″
	163 Regiment	P25, P26, P27, P29, P30	Processing tomato	E82°54′37.46″, N46°44′29.17″
	163 Regiment	P13, P14	Tomato	E82°54′47.42″, N46°44′29.71″
	163 Regiment	P17	Pumpkin	E82°54′37.59″, N46°44′26.42″
	163 Regiment	P22	Chilli	E82°54′46.90″, N46°44′28.73″
Tenth division	181 Regiment	P11	Processing tomato	E87°46′59.94″, N47°35′9.66″
		P35		
	183 Regiment	P36	Sunflower	E88°5′14.92″, N47°16′48.52″
		P40		
		P44		
	187 Regiment	P39	Sunflower	E87°59′13.70″, N47°16′44.21″
		P43		
	188 Regiment	P37	Oil sunflower	E87°43′54.42″, N47°17′9.57″
	188 Regiment	P38	Sunflower	E87°43′51.95″, N47°17′9.16″
	Fu Hai county	P92	Sunflower	E87°33′11.42″, N47°7′18.06″
Twelfth division	Ju Hu farm	P91	Processing tomato	E87°3′27.48″, N44°0′27.86″
	Wu Yi farm	P42, P45, P68	Processing tomato	E87°22′58.47″, N44°1′25.45″
	Wu Yi farm	P46	Seed watermelon	E87°21′53.22″, N44°10′22.34″
Thirteenth division	Nao Mao hu farm	P12, P15	Melon	E94°59′48.04″, N43°46′8.58″
	Nan Hu village	P90	Melon	E93°25′31.94″, N42°34′39.13″
Hetian city	La Si kui town	P53	Water melon	E79°52′54.36″, N37°10′24.70″
	La Si kui town	P60, P71	Melon	E79°53′1.22″, N37°10′23.048″
	Pi Shan county	P41	Melon	E78°17′13.66″, N37°38′59.14″
Changji city	Qi Tai county	P88, P89, P93	Sunflower	E89°34′43.76″, N44°2′50.44″
	Wu Yi farm	P54	Sunflower	E87°23′1.06″, N44°1′23.21″

### DNA extraction and PCR amplification

Total DNA was extracted from all sample stems following the modified cetyl-trimethylammonium bromide (CTAB) method described by Doyle and Doyle with minor modifications [17]. The purity of the DNA was determined by 1.0% agarose gel electrophoresis and absorption at 260 nm, and the ratios A260/А280 and A260/А230 were used to determine the presence of contaminants such as proteins, polyphenolic compounds, sugars, and lipids. The samples were diluted to 50 ng/µL for PCR amplification. The ITS and *rps*2 sequences of the samples were amplified by using the primer pairs listed in Table S1. The PCR products were purified using a universal DNA purification kit (TIANGEN, Beijing) according to the manufacturer’s instructions, cloned and inserted into pMD18-T (TaKaRa Biotech, Dalian), and sequenced at BGI Genomics Co., Ltd.

### Phylogenetic analysis

All sequences obtained from the ITS and *rps*2 regions were assembled and analyzed using Lasergene DNASTAR (version 6.0) and DNAMAN (version 6.0). Multiple-sequence alignments were performed using the program Clustal W. A phylogenetic tree was constructed based on ITS and *rps*2 sequences using the neighbor-joining method in MEGA6.0, in which samples with the same sequence from the same host in the same region were excluded. The robustness of the neighbor-joining tree was estimated using bootstrap analysis with 1000 replicates.

### ISSR assay

The genetic diversity of the collected samples was analyzed using ISSR molecular markers. A total of 100 ISSR primers from the University of British Columbia were synthetized at the BGI of Beijing and used for the study, of which 11 primers showed a high percentage of polymorphism (Table 2). The PCR amplification was carried out using a DNA engine dyad® Peltierp thermal cycler (Bio-RAD) using a 20 µL reaction mixture containing 1.0 µL DNA (50 ng), 2.2 µL 10 × PCR buffer (Mg^2+^ Plus), 1.0 µL dNTPs (2.5 mM), 0.4 µL primers (10 μm/L), 0.2 µL Taq DNA polymerase (5 U/µL, TaKaRa Biotech, Dalian), and 15.2 µL distilled water. The PCR conditions were as follows: 94 °C for 4 min, followed by 35 cycles of 94 °C for 1 min, 50.3–55 °C for 30 s, 72 °C for 90 s, and a final extension of 10 min at 72 °C.

**Table 2:** List of ISSR primers used and polymorphisms of broomrape

Sl. no.	Primer no.	Primer sequence (5′→3′)	No. of total bands	No. of polymorphic bands	Percentage of polymorphism (%)	Polymorphic information content (PIC)	Marker index (MI)
1	807	(AG)_8_ T	9	8	88.89	0.91	6.47
2	808	(AG)_8_ C	13	12	92.31	0.15	1.66
3	810	(GA)_8_ T	9	9	100.0	0.89	8.01
4	812	(GA)_8_ A	19	18	94.74	0.49	8.36
5	815	(CT)_8_ G	14	14	100.0	0.86	12.04
6	817	(CA)_8_ A	11	11	100.0	0.36	3.96
7	818	(CA)_8_ G	15	15	100.0	0.77	11.55
8	825	(AC)_8_ T	16	15	93.75	0.38	5.34
9	826	(AC)_8_ C	15	15	100.0	0.64	9.60
10	827	(AC)_8_ G	16	16	100.0	0.26	4.16
11	891	HVH(TG)_7_	16	16	100.0	0.63	10.08
Total	11		154	150	–	–	–

Y = (A, G, C, T) and R= (A, G)

The PCR products were mixed with 5 µL Roti-Load-DNA loading buffer (TaKaRa Biotech, Dalian), resolved on a 1.5% agarose gel in 0.5 × Tris-borate-EDTA (TBE) buffer, and electrophoresis was carried out with a constant voltage of 120 V for 90 min, visualized under UV light, and documented using a gel documentation and image analysis system (Universal Hood, Bio-Rad). The experiment was repeated three times, and the bands appeared consistently in all three gels, which were scored and used for the analysis.

### Data analysis

Scorable and consistently reproducible DNA fragments amplified by repeating PCR amplification with each of the selected primers were transformed into a binary scoring method of the profiles as presence (= 1) and absence (= 0), and a binary data matrix was generated for all samples. The genetic variability in the population was analyzed based on the banding pattern using parameters such as genetic diversity (Ht), genetic diversity within population of the groups (Hs), the coefficient of genetic differentiation (Gst), Shannon’s information index (I), and genetic distance analysis using POPGENE version 1.32 [16]. The UPGMA tree was generated using the Numerical Taxonomy Multivariate Analysis System (NTSYS-pc) version 2.10 [17], and bootstrap analysis was conducted using 1,000 replicates. The percentage polymorphism (P) [18], polymorphism information content (PIC) [18], marker index (MI) [19], and gene flow index (Nm) were calculated as follows: P = (K/N) × 100%, where N is the total number of amplified bands and K is the number of polymorphic bands. PIC = 1 − $\sum {P}_{i}^{2}$, in which *P*_*i*_ is the frequency of the *i* th allele. MI = K × P × PIC.

## Results

### ITS and *rps*2 amplification

To identify the collected broomrape samples, the ITS and *rps*2 were amplified. The sequences of ITS and *rps*2 were approximately 694 and 494 bp, respectively (Table S2). The ratios of variable sites to all sites for ITS and *rps*2 were 33.71% and 4.86%, respectively, and the ratios of parsimony-informative characters were 20.61% and 4.66%, respectively. This indicates that the ITS region was more variable at the parsimony-informative site than the *rps*2 region.

### Phylogeny analysis based on ITS and *rps*2 sequences

**Fig. 1:**
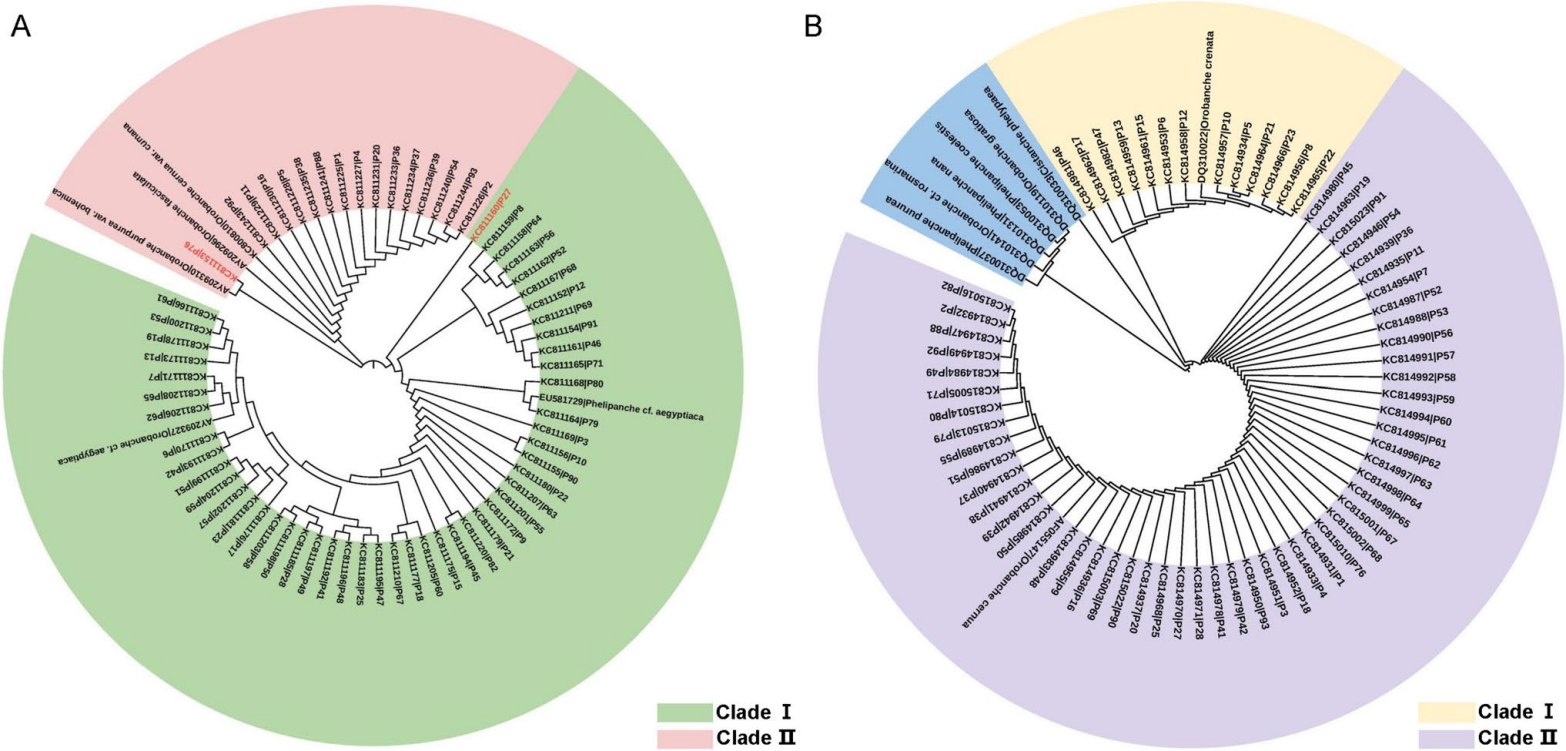
Phylogenetic trees were constructed based on the ITS (**A**) and *rps*2 (**B**) regions of broomrapes in Xinjiang by the distance-based neighbor-joining method using MEGA6. Bootstrap values were calculated from 1000 replicates of the alignment

The results showed that all collected samples were separated into two major clades based on their ITS regions, in which the first major clade consisted of 49 samples, of which two subclades were generated and had a close relationship with *O. cf. aegyptiaca*, in which the sample P27 formed a single subclade (Fig. 1A). The second major clade consisted of 16 samples with a high homology to *O. cernua* var. *cumana*, of which sample P76 had a closer relationship with *O. purpurea* var. *bohemica* (Fig. 1A). On the other hand, all the samples generated two clades based on the *rps*2 sequences, in which the 13 samples formed one clade with high homology with *O. crenata*, and the other samples formed another clade with high homology with *O. cernua* (Fig. 1B).

#### Genetic variability revealed through ISSR markers

The 11 ISSR primers were obtained by screening and generated a total of 154 bands among 93 samples, of which 150 bands were polymorphic. The number of bands generated ranged from 9 (UBC 810) to 19 (UBC 812), the PIC was from 0.15 (primer 808) to 0.91 (primer 807) with an average PIC of 0.58, and the marker index (MI) fluctuated from 1.66 for primer 808 to 12.04 for primer 815 with an average MI of 7.38. (Table 2). This indicates that broomrape has considerable polymorphism in Xinjiang Province.

To analyze the genetic diversity of the samples, 93 samples were divided into 11 geographical populations according to the distance between sampling sites and morphological differences. Population diversity analysis revealed that there was low genetic differentiation within the population because the average Nei’s gene diversity and Shannon’s information index (I) were 0.1008 and 0.1546, respectively, and the percentage of polymorphic loci was 33.18%, all of which were low (Table 3). In addition, the coefficient of genetic differentiation (GST) was 0.6281, showing that the genetic diversity of the samples was 37.19% within populations and 62.81% among the populations (Table 4), indicating that the main genetic differentiation occurred among the populations. There was less gene exchange between populations, with a gene flow index (Nm) of 0.2961 (< 1) (Table 4).

**Table 3:** Diversity parameters of 11 natural populations

Sample population	Observed number of alleles	Effective number of alleles	Nei’s (1973) gene diversity	Shannon’s Information index	The percentage of polymorphic loci (%)
Second division	1.5714	1.1826	0.1214	0.1996	57.14
Kashi city	1.3766	1.1626	0.1015	0.1598	37.66
Fourth division	1.3182	1.1700	0.1000	0.1523	31.82
Seventh division	1.1623	1.0927	0.0562	0.0849	16.23
Eighth division	1.2727	1.1610	0.0923	0.1386	27.27
Ninth division	1.3312	1.1481	0.0900	0.1406	33.12
Tenth division	1.4870	1.2491	0.1475	0.2251	48.70
Twelfth division	1.3247	1.1636	0.0988	0.1529	32.47
Thirteenth division	1.2078	1.1571	0.0867	0.1256	20.78
Hetian city	1.2208	1.1202	0.0735	0.1123	22.08
Changji city	1.3766	1.2500	0.1409	0.2085	37.66
Mean	1.3318	1.1689	0.1008	0.1546	33.18
Value on species level	1.9740	1.4070	0.2565	0.4023	97.40

**Table 4:** Summary of genetic variation statistics for all loci

Sample population	Ht	Hs	Gst	Nm
Mean	0.2710	0.1008	0.6281	0.2961

*Ht* the total gene diversity, *Hs* genetic diversity within population of the groups, *Gst* the coefficient of genetic differentiation, *Nm* gene flow index, Nm = 0.5 × (1 - Gst)/Gst

#### Cluster analysis

**Fig. 2:**
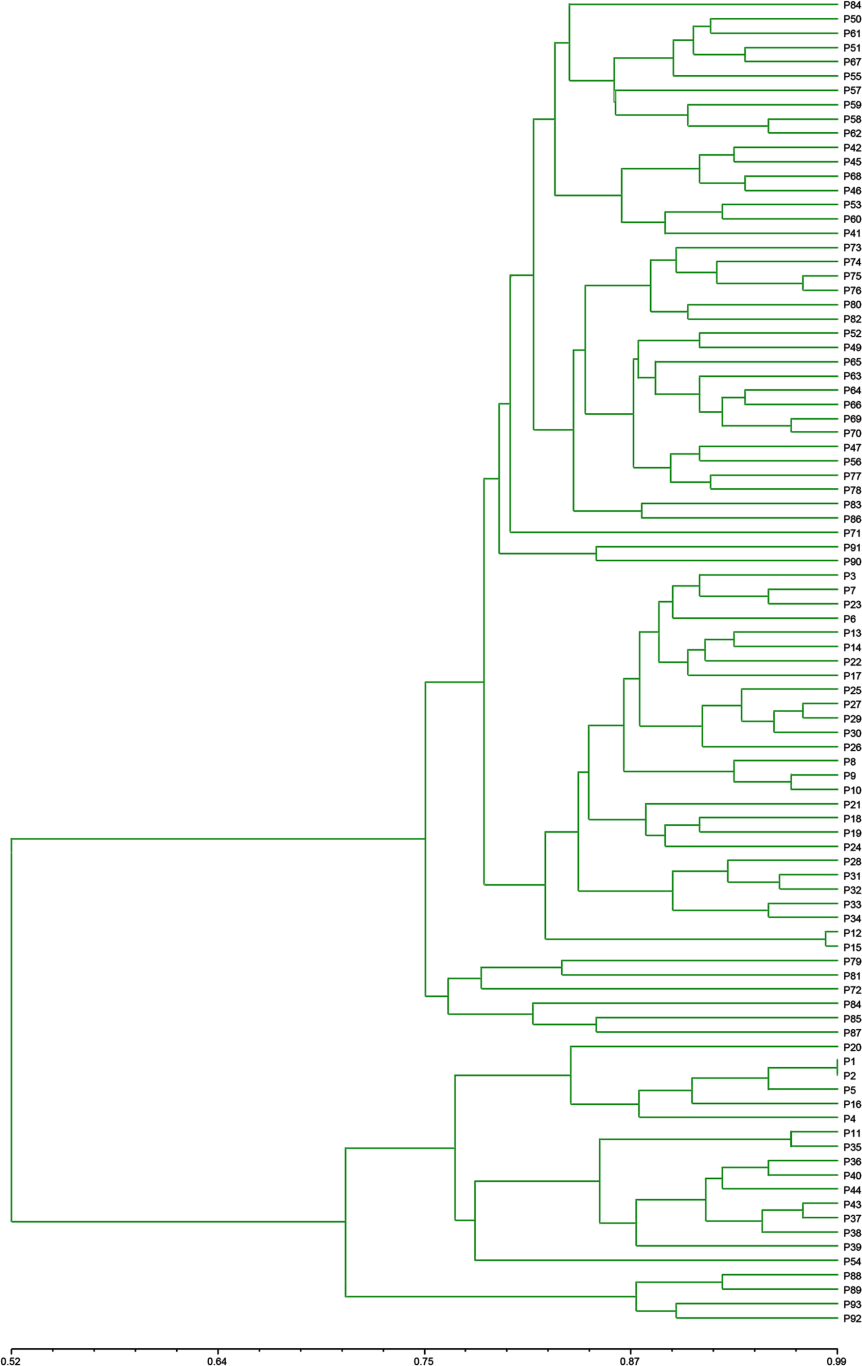
Dendrogram obtained by the UPGMA method based on ISSR markers

Similar to the phylogenetic tree constructed based on ITS sequences, all samples were clearly divided into two groups when the genetic similarity (GS) was 0.706 by UPGMA analysis based on ISSR molecular markers (Fig. 2). The samples that came from the same and near geographical origin were preferentially clustered together, except for P1 and P2, in which the first cluster included all branched columns and the second cluster included all unbranched columns. The branch group was divided into two subclusters when the GS approached 0.765. When GS approached 0.80, the upper branch of the branch group was divided into subclusters I and II, and the unbranched group was divided into four subclusters (IV, V, VI and VII). Most of the samples in subcluster I came from southern Xinjiang, and subcluster II included two samples from northern Xinjiang and eastern Xinjiang. The samples of subcluster IV mainly came from the eighth division, fifth division and Tuanjie farms. The samples of subclusters V, VI, and VII consisted of the tenth division, the twelfth division, and Qitai and Fuhai counties, respectively.

## Discussion

As a holoparasitic weed, broomrape has seriously threatened the production of economically important crops, such as melons, watermelons, processed tomatoes, and sunflower in Xinjiang in recent years. In the present study, it was found that in addition to conventional host plants, broomrape can also be parasitic on other unconventional hosts, such as cowpea, pumpkin, and pepper. In recent years, it has been found that broomrape can also be parasitic on some weeds, such as *Karelinia caspia* [22], which may be caused by the genetic variation in broomrape because the genetic diversity of species plays an important role in its adaptation to environmental changes and species evolution [23, 24]. The unique climatic conditions and greater number of hosts in Xinjiang Province may be important factors causing the genetic variation in broomrape.

Based on the phylogenetic tree constructed using ITS sequences, sample P76 had a close relationship with *O. purpurea* var. *bohemica* than *O.* cf. *aegyptiaca*, although the morphological characteristics of sample P76 were extremely similar to those of samples from the same field. However, *O. purpurea* has only been reported in a few areas of Central Europe [25]. In addition, the results of this study showed that the broomrape is not monophyletic based on the nuclear ITS region of Xinjiang Province. This is in accordance with the results of other studies based on ITS sequences and plastid marker analysis [26]. There were some small clades in the first major clade, which might be related to the hosts and their living circumstances, and had a wider host range and more changeable living circumstances than the second major clade because the host genotype might influence the evolution of parasitic plants [27–30].

Based on the phylogenetic tree constructed using *rps*2 sequences, all the samples generated one major clade. The 13 samples formed one clade with high homology to *O. crenata*, and the other samples formed another major clade with high homology to *O. cernua*. This might be related to crop rotation and the exchange of seeds in different areas because the *rps*2 gene can be transferred from members of *O. s. str*. to *Phelipanche* spp. by horizontal gene transfer via attack on the same host [31]. Although there were obvious differences in the phylogenetic relationships based on the ITS and *rps*2 regions, this implies that a close relationship occurred between *O.* cf. *aegyptiaca* and *O. cernua* to some degree. Compared with the phylogenetic tree constructed with *rps*2 sequences, the morphology of the samples agreed with the phylogenetic relationship based on the ITS region. This indicates that the evolution speed of ITS was faster than that of *rps*2.

*O.* var. *cumana* showed strong host specificity, parasitizing only sunflower and processed tomato, while *O.* cf. *aegyptiaca* parasitized a variety of crops, including processed tomatoes, melons, and melon seed, in addition to being unable to parasitize sunflower in Xinjiang Province. Interestingly, the *rps*2 genes of the two types of *O.* spp. samples showed a certain degree of homology, such as samples P2, P16, and P11 of *O. cernua* var. *cumana* and samples P82, P9, and P52 of *O* cf. *aegyptiaca* parasitizing the same host of processing tomatoes, which may indicate that processing tomatoes play a certain role in mediating gene exchange.

Broomrape had a strong ability to adapt to different climatic conditions in Xinjiang Province, while the changing climate and host diversity perhaps promoted the evolution of broomrape. The climate change stability of southern Xinjiang was stronger than that of northern Xinjiang, which may have resulted in the genetic stability of southern samples being stronger than that of northern samples. The ITS and *rps*2 values of the samples from the same region with different hosts were different, such as the samples from the 62nd regimen, 163rd regimen, and Wuyi farms. The ITS and *rps*2 genes of the samples from different regions with the same host had a close relationship, for example, samples P3, P25, P28, and P42, which may be related to the frequent dispatching of crop seeds carrying *O.* spp. seeds within different regions. This result indicated that *rps*2 of broomrape is more conserved than ITS.

The PIC plays an important role in analyzing the genetic variation [32, 33]. The PIC mean values of all primers in this study were greater than 0.25, indicating that the primers tested in this study were effective to demonstrate genetic diversity according to the research results of El-Esawi et al. [32] and Botstein et al. [34]. The primer 807 recorded the highest value of 0.91 in this study, showing high polymorphism, which is consistent with the results of genetic diversity in wheat [32]. Additionally, the values of MI were from 1.66 to 12.04 with an average of 7.38, which are in accordance with the outcomes of Akhtar et al. [35] and Ghehsareh et al. [36]. Therefore, the primers tested in this study were effective and informative. Based on the ISSR primers, a total of 150 polymorphic bands were generated, and the broomrape had higher polymorphism with the values ranging from 88.89 to 100% and a mean of 97.24%, showing that the genetic diversity of broomrape was abundant, which is similar to the genetic diversity of *O. crenata* populations in Ethiopia [37].

Similar to the phylogenetic tree constructed based on ITS sequences, all samples were clearly divided into two groups by UPGMA analysis based on ISSR molecular markers, which were correlated with the morphological characteristics. The first cluster had a wide host, and not all samples were single, with stem branching. All samples in the second cluster were single, and their host ranges were narrow. The same and nearby geographic origins were mostly gathered together, which is similar to the results of Stoyanov et al. [38]. Interestingly, sample P22 came from the 163rd regimen, and the host was chili, which was shorter than other samples of the same origin in terms of morphological characteristics; however, it clustered together with samples of the same origin. The chili may not be suitable as the host of broomrape, but the pressure of survival and breeding could oblige it to change its host range.

## Conclusion

To the best of our knowledge, this is the first study to systematically survey the molecular phylogenetic and genetic diversity of broomrape, which infests economically important crops and causes serious yield losses in Xinjiang [37, 38]. This study indicated that broomrape exhibits rich genetic diversity and that high genetic variation exists among the populations in Xinjiang. Therefore, in addition to control measures in areas where broomrape occurs, it is also important to strengthen quarantine management and prevent genetic exchanges between different broomrape populations. The results of the present study provide a theoretical basis for the identification and classification of broomrape and cultivation of resistant host varieties.

## Supplementary Information

Below is the link to the electronic supplementary material.

Supplementary material 1 (XLSX 9.9 kb)

Supplementary material 2 (XLSX 11.6 kb)

## Data Availability

The datasets analyzed during the present study are available from the corresponding author upon reasonable request.

## References

[1] Genovese C, D’Angeli F, Attanasio F, Caserta G, Scarpaci KS, Nicolosi D (2021) Phytochemical composition and biological activities of *Orobanche crenata* Forssk.: a review. Nat Prod Res 35:4579–4595. 10.1080/14786419.2020.1739042 32162541 10.1080/14786419.2020.1739042

[2] Fernández-Aparicio M, Masi M, Cimmino A, Evidente A (2021) Effects of benzoquinones on radicles of Orobanche and Phelipanche Species. Plants (Basel) 10:746. 10.3390/plants10040746 33920368 10.3390/plants10040746 PMC8070214

[3] Parker C (2009) Observations on the current status of Orobanche and Striga problems worldwide. Pest Manag Sci 65:453–459. 10.1002/ps.1713 19206075 10.1002/ps.1713

[4] Vurro M (2023) Are root parasitic broomrapes still a good target for bioherbicide control? Pest Manag Sci. 10.1002/ps.7360 36641632 10.1002/ps.7360

[5] Ito S (2023) Recent advances in the regulation of root parasitic weed damage by strigolactone-related chemicals. Biosci Biotechno Biochem 87:247–255. 10.1093/bbb/zbac208 10.1093/bbb/zbac208 36610999

[6] Aly R, Matzrafi M, Bari VK (2021) Using biotechnological approaches to develop crop resistance to root parasitic weeds. Planta 253:97. 10.1007/s00425-021-03616-1 33844068 10.1007/s00425-021-03616-1

[7] Aly R (2007) Conventional and biotechnological approaches for control of parasitic weeds. In Vitro Cell Dev Biol Plant 43:304–317. 10.1007/s11627-007-9054-5

[8] En-Nahli Y, Hejjaoui K, Mentag R, Es-Safi NE, Amri M (2023) Large field screening for resistance to broomrape (*Orobanche crenata* Forsk.) In a global lentil diversity panel (GLDP) (*Lens culinaris* Medik). Plants (Basel) 12:2064. 10.3390/plants12102064 37653981 10.3390/plants12102064 PMC10222529

[9] Calderón-González Á, Pérez-Vich B, Pouilly N, Boniface MC, Louarn J, Velasco L, Muños S (2023) Association mapping for broomrape resistance in sunflower. Front Plant Sci 13:1056231. 10.3389/fpls.2022.1056231 36714707 10.3389/fpls.2022.1056231 PMC9875907

[10] Pérez-Vich B, Akhtouch B, Knapp SJ, Leon AJ, Velasco L, Fernández-Martínez JM, Berry ST (2004) Quantitative trait loci for broomrape (*Orobanche Cumana* Wallr.) Resistance in sunflower. Theor Appl Genet 109(1):92–102. 10.1007/s00122-004-1599-7 14968309 10.1007/s00122-004-1599-7

[11] Duriez P, Vautrin S, Auriac MC et al (2019) A receptor-like kinase enhances sunfower resistance to *Orobanche Cumana*. Nat Plants 5:1211–1215. 10.1038/s41477-019-0556-z 31819219 10.1038/s41477-019-0556-z

[12] Cui H, Wang N, Long X, An K, Hou M, Cui W (2020) Review of the species, hazard and management status of *Orobanche* L. in Xinjiang. Plant Quarantine 34:20–24. 10.19662/j.cnki.issn1005-2755.2020.03.004

[13] Brownstein CD, Meyer DL, Fabbri M, Bhullar BS, Gauthier JA (2022) Evolutionary origins of the prolonged extant squamate radiation. Nat Commun 13(1):7087. 10.1038/s41467-022-34217-5 36446761 10.1038/s41467-022-34217-5 PMC9708687

[14] Hristova E, Stoyanov K, Gevezova M, Denev I (2011) Application of ISSR methods in studying broomrape’s (Orobanchaceae) biodiversity in Bulgaria. Biotechnol Biotechnol 25:2248–2253. 10.5504/BBEQ.2011.0024

[15] Piwowarczyk R, Schneider AC, Góralski G, Kwolek D, Denysenko-Bennett M, Burda A, Ruraż K, Joachimiak AJ, Pedraja ÓS (2021) Phylogeny and historical biogeography analysis support caucasian and Mediterranean centers of origin of key holoparasitic Orobancheae (Orobanchaceae) lineages. PhytoKeys 174:165–194. 10.3897/phytokeys.174.62524 33776529 10.3897/phytokeys.174.62524 PMC7979677

[16] Abd El-Fatah BES, Nassef DMT (2020) Inheritance of faba bean resistance to Broomrape, genetic diversity and QTL mapping analysis. Mol Biol Rep 47:11–32. 10.1007/s11033-019-05101-1 31584142 10.1007/s11033-019-05101-1

[17] Doyle J, Doyle JL (1987) Arapid DNA isolation method for small quantities of fresh tissues. Phytochem Bul 19:11–15

[18] Rohlf FJ (2000) NTSYS-pc: numerical taxonomy and multivariate analysis system. Version 2.1. Exeter Software, New York

[19] Yeh FC, Boyle TYZ, Xiyan JM (1999) POPGENE: Microsoft window-based freeware for population genetic analysis version 1.32. University of Alberta, Edmonton

[20] Ma Q, Hu L, Xi H, Yao Z, Wang P, Zhao S, Zhang X (2023) First report of *Karelinia caspia* (pall.) Less as a new host of *Orobanche Cumana* Wallr. In Xinjiang, China. Plant Dis. 10.1094/PDIS-05-23-0988-PDN38127629

[21] Edelman NB, Mallet J (2021) Prevalence and adaptive impact of introgression. Annu Rev Genet 55:265–283. 10.1146/annurev-genet-021821-020805 34579539 10.1146/annurev-genet-021821-020805

[22] Kelly M (2019) Adaptation to climate change through genetic accommodation and assimilation of plastic phenotypes. Philos Trans R Soc Lond B Biol Sci 374(1768):20180176. 10.1098/rstb.2018.0176 30966963 10.1098/rstb.2018.0176 PMC6365860

[23] Yousefi AR, Jamshidi K, Oveisi M, Karimojeni H, Pouryosef M (2013) First report of *Orobanche purpurea* on *Achillea Wilhelmsii* in Iran. Plant Dis 97:694. 10.1094/PDIS-08-12-0750-PDN 30722183 10.1094/PDIS-08-12-0750-PDN

[24] Schneeweiss GM, Colwell A, Park JM, Jang CG, Stuessy TF (2004) Phylogeny of holoparasitic *Orobanche* (Orobanchaceae) inferred from nuclear ITS sequences. Mol Phylogenet Evol 30:465–478. 10.1016/s1055-7903(03)00210-0 14715236 10.1016/s1055-7903(03)00210-0

[25] Bendaoud F, Kim G, Larose H, Westwood JH, Zermane N, Haak DC (2022) Genotyping-by-sequencing analysis of *Orobanche crenata* populations in Algeria reveals genetic differentiation. Ecol Evol 12:e8750. 10.1002/ece3.8750 35356582 10.1002/ece3.8750 PMC8948082

[26] Gibson AK, Baffoe-Bonnie H, Penley MJ, Lin J, Owens R, Khalid A, Morran LT (2020) The evolution of parasite host range in heterogeneous host populations. J Evol Biol 33:773–782. 10.1111/jeb.13608 32086852 10.1111/jeb.13608 PMC7275899

[27] Conn CE, Bythell-Douglas R, Neumann D, Yoshida S, Whittington B, Westwood JH, Shirasu K, Bond CS, Dyer KA, Nelson DC (2015) Plant evolution. Convergent evolution of strigolactone perception enabled host detection in parasitic plants. Science 349:540–543. 10.1126/science.aab1140 26228149 10.1126/science.aab1140

[28] Sallinen S, Laine AL (2023) Short-term fitness consequences of parasitism depend on host genotype and within-host parasite community. Evolution 77:1806–1817. 10.1093/evolut/qpad090 37195704 10.1093/evolut/qpad090

[29] Park JM, Manen JF, Schneeweiss GM (2007) Horizontal gene transfer of a plastid gene in the nonphotosynthetic flowering plants *Orobanche* and *Phelipanche*. Mol Phylogenet Evol 43:974–985. 10.1016/j.ympev.2006.10.011. Orobanchaceae 17116411 10.1016/j.ympev.2006.10.011

[30] El-Esawi MA, Elashtokhy MMA, Shamseldin SAM, El-Ballat EM, Zayed EM, Heikal YM (2022) Analysis of genetic diversity and phylogenetic relationships of wheat (*Triticum aestivum* L.) genotypes using phenological, molecular and DNA barcoding markers. Genes (Basel) 14(1):34. 10.3390/genes14010034 36672774 10.3390/genes14010034 PMC9858705

[31] Peng JH, Lapitan NL (2005) Characterization of EST-derived microsatellites in the wheat genome and development of eSSR markers. Funct Integr Genomics 5(2):80–96. 10.1007/s10142-004-0128-8 15650880 10.1007/s10142-004-0128-8

[32] Botstein D, White RL, Skolnick M, Davis RW (1980) Construction of a genetic linkage map in man using restriction fragment length polymorphisms. Am J Hum Genet 32:314–331 6247908 PMC1686077

[33] Akhtar N, Hafiz IA, Hayat MQ, Potter D, Abbasi NA, Habib U, Hussain A, Hafeez H, Bashir MA, Malik SI (2021) ISSR-based genetic diversity assessment of Genus *Jasminum* L. (Oleaceae) from Pakistan. Plants (Basel) 10(7):1270. 10.3390/plants10071270 34206638 10.3390/plants10071270 PMC8308950

[34] Ghasemi GM, Salehi H, Khosh-Khui M, Niazi A (2015) Application of ISSR markers to analyze molecular relationships in Iranian jasmine (*Jasminum* spp.) accessions. Mol Biotechnol 57(1):65–74. 10.1007/s12033-014-9802-9 25189463 10.1007/s12033-014-9802-9

[35] Belay G, Tesfaye K, Hamwieh A, Ahmed S, Dejene T, de Oliveira Júnior JOL (2020) Genetic diversity of *Orobanche crenata* populations in Ethiopia using microsatellite markers. Int J Genomics 2020:3202037. 10.1155/2020/3202037 32855960 10.1155/2020/3202037 PMC7442992

[36] Stoyanov K, Gevezova M, Denev I (2012) Identification of ISSR markers for studying the biodiversity of Bulgarian representatives of genus *Orobanche* Subsection Minores. Biotechnol Biotec Eq. 26:2743–2749. 10.5504/bbeq.2011.0139

[37] Zhang L, Cao X, Yao Z, Dong X, Chen M, Xiao L, Zhao S (2022) Identification of risk areas for *Orobanche Cumana* and *Phelipanche Aegyptiaca* in China, based on the major host plant and CMIP6 climate scenarios. Ecol Evol 12:e8824. 10.1002/ece3.8824 35462975 10.1002/ece3.8824 PMC9018459

[38] He W, Li Y, Luo W, Zhou J, Zhao S, Xu J (2022) Herbicidal secondary metabolites from *Bacillus velezensis* JTB8-2 against *Orobanche Aegyptiaca*. AMB Express 12:52. 10.1186/s13568-022-01395-w 35524860 10.1186/s13568-022-01395-w PMC9079202

